# Méningo-encéphalite tuberculeuse révélée par des troubles psychiatriques: à propos d’un cas

**DOI:** 10.11604/pamj.2017.27.206.12811

**Published:** 2017-07-20

**Authors:** Khalid Mouhadi, Tarik Boulahri, Abdelhadi Rouimi

**Affiliations:** 1 Faculté de Médecine et de Pharmacie, Marrakech, Maroc; 2 Service de Neurologie, Hôpital Militaire Moulay Ismail, Meknès, Maroc

**Keywords:** Tuberculose, méningo-encéphalite, trouble psychiatrique, Tuberculosis, meningoencephalitis, psychiatric disorder

## Abstract

La méningo-encéphalite tuberculeuse est une pathologie assez fréquente dans les pays endémiques, et représente la forme la plus sévère de la tuberculose. L’échec thérapeutique est fréquent à cause du retard diagnostique. Ce retard est essentiellement dû au grand polymorphisme clinique et notamment en raison des formes trompeuses. Ce travail expose le cas clinique rare d’une méningite tuberculeuse se présentant sous forme d’un tableau psychiatrique typique au cours de sa phase prodromique.

## Abstract (en)

Tuberculous meningoencephalitis is fairly frequent in endemic countries and it is the most severe form of tuberculosis. Therapeutic failure is common because of diagnostic delay. This delay is primarily due to a wide clinical polymorphism and, in particular, to misleading forms. We here report a rare clinical case of tuberculous meningitis in a patient in prodromal phase of psychosis.

## Introduction

La méningo-encéphalite tuberculeuse est une urgence diagnostique et thérapeutique. Elle associe au tableau méningé, une atteinte encéphalique avec des troubles de la conscience, une perturbation des fonctions supérieures et des signes de focalisation neurologique [1]. Les symptômes psychiatrique sont peu rapportés, et passent toujours au deuxième plan derrière les tableaux neurologique et infectieux. Ce travail expose le cas d’une méningo-encéphalite tuberculeuse de forme trompeuse, se manifestant initialement par des troubles psychiatriques typique, ayant retardé le diagnostic.

## Patient et observation

Il s’agit d’un jeune homme de 36 ans, droitier, célibataire, surveillant pénitentiaire, sans antécédents médico-chirurgicaux ou psychiatriques personnels ou familiaux notables, ni de conduites addictives connues. Il s’est présenté en consultation psychiatrique accompagné de son frère pour un syndrome dépressif classique, avec une humeur dépressive, une tendance à l’isolement, une irritabilité, une perte d’appétit, une asthénie et une hypersomnie. A coté de ce tableau dépressif, l’entretien avait relevé la présence de céphalées modérées généralisées.

**Figure 1:**
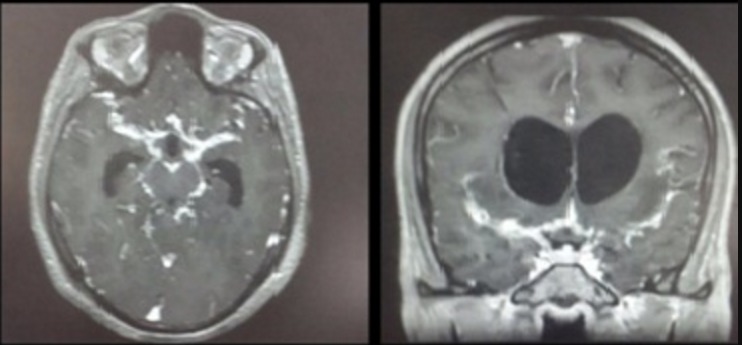
IRM cérébrale coupe axiale et coronale en séquence pondéré T1 injectée: montrant un épaississement et un rehaussement des leptoméninges prédominant au niveau des vallées sylviennes associés à une hydrocéphalie

Le diagnostic d’épisode dépressif caractérisé a été évoqué, et le patient fut mis sous antidépresseur (Tianéptine 12,5 mg: 3 comprimés /jours). Après 3 semaines de traitement, L’évolution a été marquée par l’apparition d’une désinhibition et exhibitionnisme en plus de la symptomatologie initiale faisant suspecter un virage maniaque, ce qui a motivé son admission dans notre formation. Une semaine après son hospitalisation, le patient a présenté une symptomatologie neurologique faite d’une chute de la paupière supérieure droite avec diplopie, des vomissements en jet et une augmentation de l’intensité des céphalées. De ce fait, un avis neurologique a été demandé et l’examen neurologique avait trouvé un syndrome méningé fébrile, une diparésie faciale et une paralysie complète du nerf oculomoteur commun droit. Le scanner cérébral avait montré une hydrocéphalée isolée. L’IRM cérébrale montrait sur les séquences pondérés T1 après injection de gadolinium un épaississement et un rehaussement des leptoméninges prédominant au niveau des valleés sylviennes associés à une hydrocéphalie (Figure 1).

**Figure 2:**
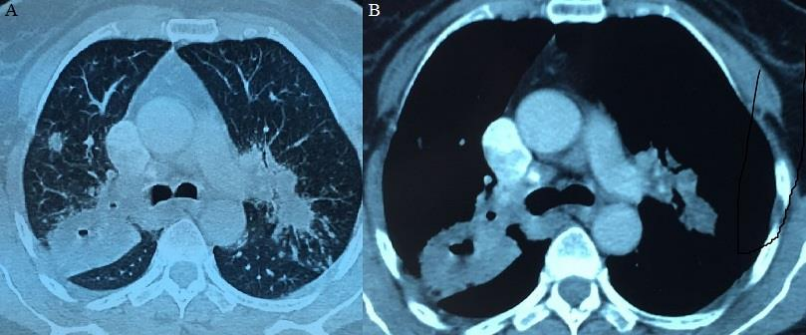
TDM thoracique coupe axiale fenêtre médiastinale (A) et fenêtre parenchymateuse; (B): présence de plusieurs foyers de condensation pulmonaires associées à des opacités nodulaires et des adénopathies médiastinales bilatérales

L’étude du LCR révélait une hyperprotéinorachie à 2,24 g/L, une glycorachie à 0,2g/L pour une glycémie de 0,95 g/L soit un rapport de 0,21%, une hypochlorurorachie à 113 mmol/L et une pléiocytose à 235 leucocytes/mm3 avec une lymphocytose à 80 %. L’examen direct et les cultures étaient négatifs. La PCR dans le LCR était positive au Mycobactérium tuberculosis. Le scanner thoracique avait montré la présence des foyers de condensation pulmonaires associées à des opacités nodulaires et des adénopathies médiastinales bilatérales (Figure 2).

L’hémogramme révélait une lymphopénie à 780/UL. L’ionogramme sanguin montrait une hyponatrémie à 126 mmol/L et la CRP était à 26mg/L, l’IDR (intra dermoréaction à la tuberculine) était négative ainsi que la recherche de BK dans les crachats et les urines. L’examen ophtalmologique était sans anomalie et la sérologie VIH négative.

Le diagnostic d’une méningo-encéphalite tuberculeuse a été retenu, et le patient a été mis sous traitement anti-bacillaires à base d’Ethombitol, Rifampicine , Isoniazide et de Pyrazinamide pendant deux mois suivi d’ une bithérapie associant Isoniazide et Rifampicine pendant 10 mois ainsi qu’une corticothérapie à base de prédnisolone 1mg/k/j pendant les deux premiers mois du traitement . L’évolution a été marquée, d’une part, par une disparition progressive des symptômes psychiatriques et neurologiques à l’exception de la diparésie faciale qui a persisté malgré les séances de rééducation, et d’autre part, par un effacement des lésions à L’IRM cérébrale de contrôle.

## Discussion

La tuberculose continue d’être une maladie endémique dans plusieurs pays africains [2]. Au Maroc, le nombre de nouveaux cas de tuberculose est estimé entre 27000 et 28000 par an selon les chiffres du «plan national d’accélération de la réduction de l’incidence de la tuberculose 2013-2016» du ministère de la santé. Le pourcentage de l’atteinte du système nerveux central (SNC) varie selon les auteurs entre 1 et 30% [3, 4]. Il peut s’agir d’une méningite et/ou d’une méningo-encéphalite, d’une atteinte médullaire ou radiculaire, d’une lésion expansive intracrânienne ou intra médullaire [4].

Classiquement, la phase prodromique de la tuberculeuse du SNC se caractérise par, une fièvre modérée, des sueurs, une perte d’appétit, une tendance à la somnolence diurne, des céphalées, une irritabilité et plus rarement des troubles de l´humeur et du comportement sans pour autant constituer un véritable tableau psychiatrique [3, 5]. Il s’agit donc de symptômes non spécifiques, rendant difficiles le diagnostic précoce. Ce n’est qu’au cours de la phase d’état que le tableau clinique devient plus clair, avec l’apparition des symptômes confusionnels et d’un syndrome neuro-méningé, avec une altération de l’état général.

Selon le British medical researche council [5], des critères cliniques de sévérité de la méningite tuberculeuse ont été définis, et trois stades ont été différenciés; le premier correspond à la phase prodromique où il n’y a pas de symptômes confusionnels ou neurologiques, avec une mortalité inférieure à 10%, alors qu’elle celle-ci augmente à 20 % et jusqu’à 70% dans les stades 2 et 3 qui correspondent à la phase d’état [6–8]. Cette forte mortalité et la fréquence des séquelles neurologiques, montrent l’importance du diagnostic précoce, notamment durant la phase prodromique. Les raisons les plus connues pour un diagnostic tardif sont la présence de symptômes somatiques bénins attribués à tort à une infection systémique, et la mauvaise interprétation des anomalies du LCR (liquide céphalo-rachidien) attribuant à tort la méningite à une origine bactérienne non tuberculeuses [9].

Dans le cas présent, la phase prodromique a été caractérisée par la prédominance d’un trouble dépressif typique, avec une évolution trompeuse au début, orientant vers un virage maniaque induit par l’antidépresseur prescrit. La présence de céphalées est habituelle dans les syndromes dépressifs, et n’alerte pas toujours les psychiatres, sauf en cas de leur sévérité ou leur persistance. Mais dans ce cas, ces céphalées étaient le seul signe avant coureur de l’atteinte cérébro-méningée, alors que la fébricule passait inaperçue avant l’hospitalisation.

La disparition des manifestations psychiatriques sous traitement antibacillaire seul, administré selon un schéma thérapeutique proposé par l’OMS [7], permet de conclure à leur origine infectieuse. A ce jour, aucun cas similaire n’a été rapporté dans la littérature. Cela montre l’intérêt de donner beaucoup d’importance aux symptômes physiques, même minimes, et les rechercher activement devant tout tableau psychiatrique, afin de dépister à temps une étiologie organique sous jacente, et la prendre en charge rapidement. Le pronostic vital étant fortement engagé après la phase prodromique, celle-ci mérite d’être minutieusement étudiée.

## Conclusion

L’expression clinique de la méningo-encéphalite tuberculeuse est très polymorphe, et le mode de début peut être trompeur. La phase prodromique peut se présenter sous forme d’un tableau psychiatrique authentique, avec des symptômes physiques minimes. La mortalité élevée et la fréquence des séquelles neurologiques imposent une attention particulière à ces symptômes non spécifiques.

## Conflits d’intérêts

Les auteurs ne déclarent aucun conflit d’intérêt.
